# Integrating Genetics And Metabolomics Reveals Putative Mechanisms For Hippuric acid Delaying Kidney Aging

**DOI:** 10.1093/geroni/igaf122.3166

**Published:** 2025-12-31

**Authors:** Yaxin Li, Bangwei Chen, Li Luo, Shengyin Zeng, Lei Ruan, Shida Zhu, Cuntai Zhang, Tao Li

**Affiliations:** BGI Genomics, Shenzhen, Guangdong, China (People’s Republic); BGI Genomics, Shenzhen, Guangdong, China (People’s Republic); BGI Genomics, Shenzhen, Guangdong, China (People’s Republic); BGI Genomics, Shenzhen, Guangdong, China (People’s Republic); Huazhong University of Science and Technology, Wuhan, Hubei, China (People’s Republic); BGI Genomics, Shenzhen, Guangdong, China (People’s Republic); Huazhong University of Science and Technology, Wuhan, Hubei, China (People’s Republic); BGI Genomics, Shenzhen, Guangdong, China (People’s Republic)

## Abstract

Increased life expectancy has led to a rise in age-related diseases. Hippuric acid (HPA) is a potential biomarker for aging. We aim to examine the associations between plasma HPA and biological aging of different organs, and identify the influence factor by integrating genetic and metabolomic data. In 777 healthy participants, Klemera-Doubal method was used to estimate biological aging and the discrepancy (Δage) between biological age and chronological age. Multiple linear regression was used to assess the relationship between plasma HPA and organ age or Δage (heart, kidney, liver, vascular). Genetic and metabolomic data were integrated to identify factors influencing HPA and kidney aging, and Mendelian randomization (MR) analysis was performed to examine causality. Plasma HPA levels were positively associated with chronological age and liver Δage, but negatively correlated with Δage of heart, kidney (β: -0.794, 95% CI: -1.352, -0.235), and vascular. The MR analysis further support a causal relationship between the HPA and kidney Δage (β: -0.009, 95% CI: -0.015, -0.003). What’s more, the INPP4B and STEAP2 gene regions were identified as modulating HPA’s effects on kidney aging. Metabolomic analysis showed that specific pathways, including alanine, aspartate, and glutamate metabolism; arginine biosynthesis; and glyoxylate and dicarboxylate metabolism, may play a role in HPA against kidney aging. In conclusion, HPA has distinct associations with different organic aging, especially acting as a protective biomarker for kidney aging. The association is influenced by both genetic and metabolic factors. HPA may provide for early intervention in kidney aging.

